# Hartree–Fock interaction in superconducting condensate fractals

**DOI:** 10.3762/bjnano.16.150

**Published:** 2025-12-04

**Authors:** Edward G Nikonov, Yajiang Chen, Mauro M Doria, Arkady A Shanenko

**Affiliations:** 1 HSE University, 101000 Moscow, Russia; 2 Meshcheryakov Laboratory of Information Technologies, Joint Institute for Nuclear Research, Dubna, Russia; 3 Zhejiang Key Laboratory of Quantum State Control and Optical Field Manipulation, Department of Physics, Zhejiang Sci-Tech University, 310018 Hangzhou, China; 4 Instituto de Física, Universidade Federal do Rio de Janeiro, 21941-972 Rio de Janeiro, Brazil

**Keywords:** Fibonacci chain, fractal superconductivity, Hartree–Fock interaction, quasicrystal

## Abstract

It is well known that the Hartree–Fock (HF) interaction does not alter observables in conventional superconductors as its effect is mainly reduced to a chemical potential shift. Deviations from this behavior can only arise in situations of translational symmetry breaking, for example, caused by the presence of external fields that induce spatial variations of the order parameter and electron density. We demonstrate that this scenario changes fundamentally in quasicrystalline systems, where the intrinsic lack of translational symmetry leads to a fractal spatial distribution of the superconducting condensate and electron density. By investigating a Fibonacci chain as a prototype quasicrystal, we numerically solve the Bogoliubov–de Gennes equations and show that, beyond the half-filling, the HF potential significantly enhances the self-similar spatial oscillations of the order parameter while simultaneously reducing its average value and altering its critical exponent. Consequently, the critical temperature is suppressed; for our chosen microscopic parameters, this suppression can reach up to 20%. Therefore, an accurate analysis of condensate distribution and related quantities in quasicrystalline superconductors requires the comparison of results obtained with and without the HF interaction.

## Introduction

It is well known, dating back to the classical book by de Gennes [1], that, in conventional superconducting materials, the Hartree–Fock (HF) interaction merely reduces to a shift of the chemical potential, as the observables are not affected due to translational invariance. Hence, the HF field is a kind of “spectator” that defines the single-particle states and chemical potential but does not act on the pair formation and, thus, can be neglected, as in the standard formulation of the BCS model [2–3]. Nevertheless, the HF potential cannot be neglected in the presence of external fields [4], such as impurity potentials [5–6], quantum confinement in nanoscale superconductors [7], and potential barriers at interfaces [8]. Such external fields break the translational invariance, which is the condition for the HF field to make a contribution to the formation of the superconducting condensate.

This raises an interesting question about systems that exhibit an intrinsic lack of translational invariance even in the absence of any applied field. Among those are quasicrystals, which were first discovered in 1984 [9–11]. Quasicrystals exhibit long-range orientational order, such as the fivefold symmetry in Al_86_Mn_14_ alloys [9–10], but lack the translational invariance [11]. The superconductivity of quasicrystals was established in 2018 with the discovery of superconducting signatures in an Al–Zn–Mg alloy below a critical temperature of *T*_c_ ∼ 0.05 K [12]. More recently, in 2024 and 2025, much higher critical temperatures of *T*_c_ ∼ 1 K and *T*_c_ ∼ 5.47 K were reported in van der Waals-layered dodecagonal quasicrystals Ta_1.6_Te [13] and in a monoclinic approximant to the decagonal quasicrystal Al_13_Os_4_[14], respectively.

Experimental observations of the superconductivity in quasicrystals ignited big interest regarding many open problems related to the superconducting condensate in quasiperiodic systems. Most of the recent results were obtained for a superconducting Fibonacci chain, being a simplified one-dimensional model for superconducting quasicrystals [15]. Using this model, researchers explored a range of phenomena in quasiperiodic systems, including proximity effects in quasicrystal–metal hybrids [16–18], enhanced superconductivity from staggered hopping amplitudes [19], and the interplay between the Josephson effect and quasiperiodicity [20]. The model has also been used to investigate topological superconductivity [21] and anomalous local critical temperatures (at the left end, at the right end, and at the chain center) in quasiperiodic chains [22]. These investigations demonstrate that the spatial distribution of the superconducting condensate in quasiperiodic chains exhibits a distinct fractal character, with significant oscillations of the order parameter along the system. A similar fractal inhomogeneous distribution of the pair condensate has been calculated for Penrose and Ammann–Beenker tilings [23], well-known representations of two-dimensional quasicrystals.

Recent studies confirm that the superconducting condensate in quasiperiodic systems possesses a highly nontrivial spatial structure. This finding naturally raises the question of how sensitive the theoretical predictions for quasicrystalline superconductors are to the inclusion of the HF potential in the fundamental microscopic equations. Our work addresses this open problem through an investigation of the superconducting Fibonacci chain, a standard prototype for quasiperiodic systems.

## Bogoliubov–de Gennes Equations for Superconducting Fibonacci Chains

To investigate the superconducting properties of a Fibonacci chain, we use an attractive Hubbard model with the grand-canonical Hamiltonian (absorbing the chemical potential μ) given by [4–6,8,19,22],


[1]

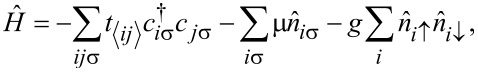



where *c_iσ_* and 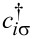 are, respectively, the annihilation and creation operators of an electron with the spin projection σ = (↑,↓) at sites *i* = 1,…,*N*, *t*_⟨_*_ij_*_⟩_ is the hopping amplitude between the nearest neighboring sites, 
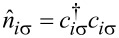
, and *g >* 0 is the on-site attractive electron–electron interaction.

Within the mean-field approximation, the Hamiltonian in Equation 1 is reduced [4] to the effective BCS–Bogoliubov Hamiltonian in the form (for the s-wave pairing):


[2]

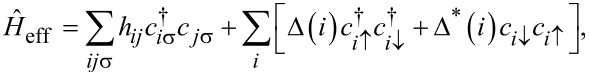



where


[3] $$h_{ij}=-t_{\langle ij\rangle }-\delta_{ij}\left[\mu -U_{\text{HF}}\left(i\right)\right],$$

with δ*_ij_* the Kronecker delta, and Δ(*i*) and *U*_HF_(*i*) the superconducting order parameter and the HF interaction potential, respectively. The latter obey the self-consistency relations


[4]

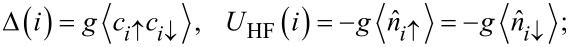



here, we exclude spin-imbalanced regimes in which 
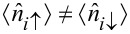
.

The effective Hamiltonian is diagonalized by applying the Bogoliubov–Valatin transformation [4],


[5] $$\left(\begin{array}{c} c_{i↑}\\ c_{i↓}^{†} \end{array}\right)=\sum_{\nu }\left(\begin{array}{cc} u_{\nu }\left(i\right) & -v_{\nu }^{*}\left(i\right)\\ v_{\nu }\left(i\right) & u_{\nu }^{*}\left(i\right) \end{array}\right)\left(\begin{array}{c} \gamma_{\nu ↑}\\ \gamma_{\nu ↓}^{†} \end{array}\right),$$

where *u*_ν_(*i*) and *v*_ν_(*i*) are, respectively, the particle-like and hole-like quasiparticle (bogolon) wavefunctions, and γ_νσ_ and 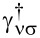 are the annihilation and creation operators for bogolon state ν, σ, respectively. The quasiparticle wave functions obey the Bogoliubov–de Gennes equations


[6] $$\begin{array}{c} \sum_{j}h_{ij}u_{\nu }\left(j\right)+\Delta \left(i\right)v_{\nu }\left(i\right)=\varepsilon_{\nu }u_{\nu }(i),\\ \Delta^{*}\left(i\right)u_{\nu }\left(i\right)-\sum_{j}h_{ij}^{*}v_{\nu }\left(j\right)=\varepsilon_{\nu }v_{\nu }\left(i\right), \end{array}$$

where ε_ν_ is the quasiparticle energy. As a result of the diagonalization, one obtains


[7] $$\langle \gamma_{\nu ↑}^{†}\gamma_{\nu ↑}\rangle =\langle \gamma_{\nu ↓}^{†}\gamma_{\nu ↓}\rangle =f_{\nu },\;\langle \gamma_{\nu ↑}\gamma_{\nu ↓}\rangle =0,$$

where *f*_ν_ is the Fermi–Dirac distribution of bogolons with the quasiparticle energy ε_ν_. The quantum number ν enumerates the quasiparticle states in ascending energy order. In our study, we employ the open-boundary conditions [5,8,22] for the quasiparticle wavefunctions *u*_ν_(*i*) and *v*_ν_(*i*), which corresponds to the physical scenario of electrons being quantum-confined within the chain.

When using Equation 5 and Equation 7, the self-consistency relations given by Equation 4 are represented in the form


[8] $$\begin{array}{c} \Delta \left(i\right)=g\sum_{\nu }u_{\nu }\left(i\right)v_{\nu }^{*}\left(i\right)\left[1-2f_{\nu }\right],\\ U_{\text{HF}}\left(i\right)=-g\sum_{\nu }\left[{\left|u_{\nu }\left(i\right)\right|}^{2}f_{\nu }+{\left|v_{\nu }\left(i\right)\right|}^{2}\left(1-f_{\nu }\right)\right]. \end{array}$$

In addition, the averaged occupation number of electrons is given by


[9] $$n_{\text{e}}=\frac{1}{N}\sum_{i\sigma }\langle {\hat{n}}_{i\sigma }\rangle =2\sum_{i\nu }\left[f_{\nu }{\left|u_{\nu }\left(i\right)\right|}^{2}+\left(1-f_{\nu }\right){\left|v_{\nu }\left(i\right)\right|}^{2}\right],$$

which defines the chemical potential μ. The summation in Equation 8 and Equation 9 is over the quasiparticle species with positive energies. In addition, the summation in Δ(*i*) is limited to the states in the Debye window around the Fermi level, that is, 0 ≤ ε_ν_ ≤ ℏω_D_. However, in the current study, we assume that ℏω_D_ is much larger than the half-bandwidth. This assumption renders the Debye energy constraint ineffective as all solutions of the Bogoliubov–de Gennes (BdG) equations with positive quasiparticle energies consequently fall within the Debye window.

The self-consistent calculation procedure follows the same protocol as for the periodic Hubbard model. First, we solve the BdG equations (Equation 6) using an initial guess for μ, Δ(*i*), and *U*_HF_(*i*). Second, using the resulting quasiparticle energies and wave functions, we compute new values for Δ(*i*) and *U*_HF_(*i*) from Equation 8. Third, we adjust μ to achieve the desired average occupation number *n*_e_ from Equation 9. The new values of μ, Δ(*i*), and *U*_HF_(*i*) are then reinserted into the BdG equations, and the entire procedure is repeated until convergence is achieved. The calculation is considered converged when the relative changes in the order parameter and the HF field are below 10^−7^.

To model quasicrystal superconducting properties, as the first step, we consider a finite Fibonacci sequence (Fibonacci approximant) *S_n_*, with *n* being the characteristic sequence number [15]. This is a sequence of symbols “A” and “B”, which is the concatenation of sequences *S_n−1_* and *S_n−2_*, that is, *S_n_* = [*S_n−1_*, *S_n−2_*], where *S*_1_ = [B] and *S*_2_ = [A] include only one symbol [15]. Based on this Fibonacci rule, we have *S*_3_ = [AB], *S*_4_ = [ABA], *S*_5_ = [ABAAB], *S*_6_ = [ABAABABA] and so on. The number of symbols in *S_n_* is *F_n_*, and {*F*_1_, *F*_2_, *F*_3_, *F*_4_, *F*_5_,…} = {1, 1, 2, 3, 5,…}, which are the Fibonacci numbers. We then map this sequence onto a physical lattice using the off-diagonal model. Each symbol A or B in the sequence defines the hopping parameters *t*_A_ or *t*_B_, respectively, between adjacent lattice sites. This results in a one-dimensional chain with a total of *N* = *F_n_* + 1 sites, following the well-established off-diagonal formulation of the Fibonacci model [15,19,24–25].

All energy-related quantities, that is, Δ(*i*), *U*_HF_(*i*), μ, *T*, *t*_A_, and *g*, are expressed in units of the hopping parameter *t*_B_. We set *g* = 2 and consider two different values of the Fibonacci sequence index, *n =* 12 and *n* = 13, for a more detailed illustration. Furthermore, we investigate two variants of the hopping amplitudes, namely, *t*_A_ = 0.5 and *t*_A_ = 1.5 (in units of *t*_B_). Our calculations are performed away from the half-filling as this regime was shown to produce a uniform electronic distribution in Fibonacci chains [22], where the HF potential does not alter superconducting properties. Here, we adopt an electron density of *n*_e_ = 0.5. Our qualitative conclusions are robust and not sensitive to the specific choice of these model parameters.

## Results and Discussion

**Figure 1:**
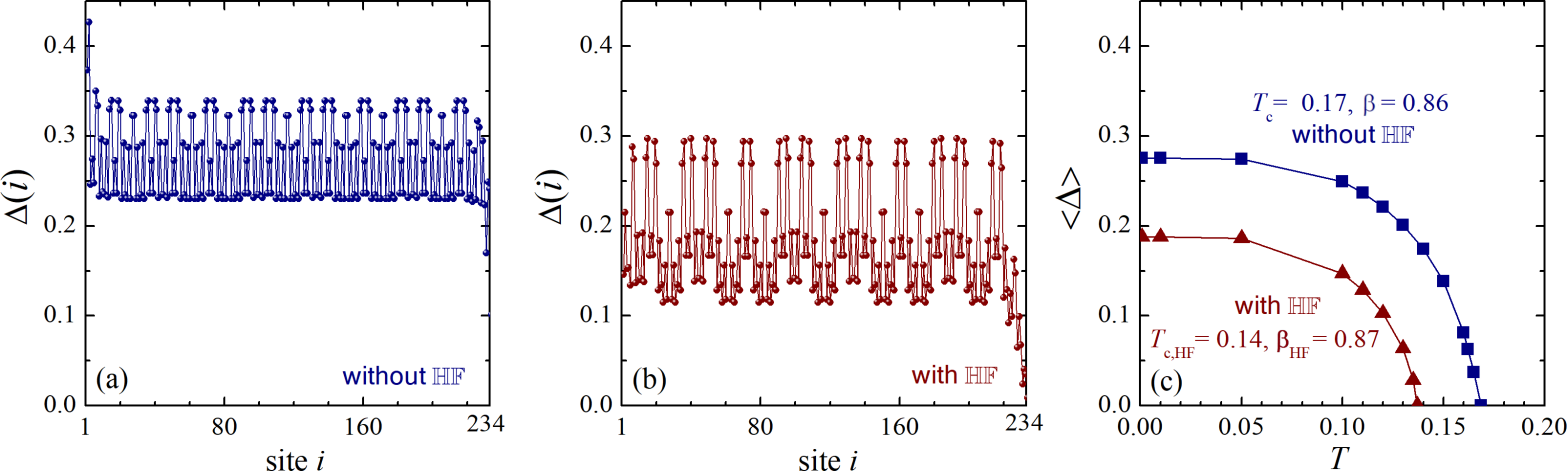
The spatial distribution of the order parameter in the Fibonacci chain with *n* = 12 and *t*_A_ = 1.5, without the HF potential (a) and with the HF potential (b). Panel (c) represents the temperature-dependence of the average order parameter in the chain center with (triangles) and without (squares) HF interaction. The critical temperatures with and without HF interaction are *T*_c,HF_ = 0.14 and *T*_c_ = 0.17; the corresponding critical exponents of the averaged order parameter are β_HF_ = 0.87 (with HF) and β = 0.86 (without HF).

Figure 1 shows results of numerically solving the BdG equations in a self-consistent manner for *n* = 12 and *t*_A_ = 1.5. In this case, *F_n=12_* = 233; consequently, the number of atomic sites in the chain is *N* = 234. In Figure 1a, one can see the spatial profile of the order parameter Δ(*i*) calculated at zero temperature by taking into account the HF interaction. The order parameter exhibits significant oscillations due to the quasiperiodic character of the system. These oscillations in the Fibonacci approximant with *n* = 12 are connected with the fractal distribution of the condensate in the infinite Fibonacci chain. In agreement with a previous investigation [22], there are three spatial regions with clearly different averages of the order parameter, namely, the left-end domain, the center of the chain, and the right-end region. The order parameter is enhanced up to 0.43 near the left end, while it is reduced to 0.16 near the right end. The average value of Δ(*i*) near the chain center (averaging in the interval from *i* = 70 to *i* = 170) is 0.28. This feature is related to the presence of three critical temperatures, that is, the left-end, the right-end, and the center (bulk) superconducting temperature, as reported in [22].

We now examine the zero-temperature order parameter for the system with the HF potential, as shown in Figure 1b. The oscillations of the order parameter are immediately apparent and are significantly more pronounced than in the system without the HF potential. In Figure 1a, the total range of the oscillations (from their minimum to their maximum in a given region) is approximately 30% of the average order parameter value, whereas in Figure 1b, this value reaches nearly 100%. Furthermore, including the HF interaction qualitatively alters the spatial distribution of the condensate near the chain edges. Specifically, the enhancement of the order parameter near the left end, which is clearly present without the HF potential, is suppressed when the HF interaction is included, as seen in Figure 1b. Concurrently, the suppression of the order parameter near the right chain end becomes even more pronounced in the system with HF interaction.

To further analyze the system, Figure 1c shows the temperature-dependence of the order parameter averaged over the center of the chain, ⟨Δ⟩ (in the interval from *i* = 80 to *i* = 160). The inclusion of HF interaction results in a significant decrease of both the order parameter in the chain center and the corresponding critical temperature. When the HF potential is included, the zero-temperature order parameter is ⟨Δ⟩*_T=0_*_,HF_ = 0.19, compared to a value of approximately 0.28 without it. The critical temperatures are *T*_c_ = 0.17 and *T*_c,HF_ = 0.14. The ratio ⟨Δ⟩*_T=0_*_,HF_/*T*_c,HF_ = 1.36 is notably smaller than the corresponding ratio without the HF field, ⟨Δ⟩*_T=0_*/*T*_c_ = 1.64. Furthermore, both values are smaller than the universal BCS prediction of Δ(0)/*T*_c_ = 1.76.

Finally, using the temperature-dependent data from Figure 1c, we calculate the critical exponent β of the order parameter near the critical temperature:


[10] $$\langle \Delta \rangle \propto \tau^{\beta },$$

where τ = 1 − *T*/*T*_c_ (or *T*_c,HF_ for the chain with the HF interaction). Our analysis shows that β = 0.86 without the HF field, while β_HF_ = 0.87 with it. These values are only slightly different. However, both of them are significantly larger than the BCS order-parameter critical exponent of 0.5. This observation agrees with previous expectations [26–27] of power-law scaling with non-standard exponents for thermodynamic properties of superconducting quasicrystals near *T*_c_. Here, we note an early investigation of another quasiperiodic one-dimensional quantum system, namely, the Ising model on a transverse applied field, which studied the phase transition occurring in its coupling parameter and related critical indexes [28].

**Figure 2:**
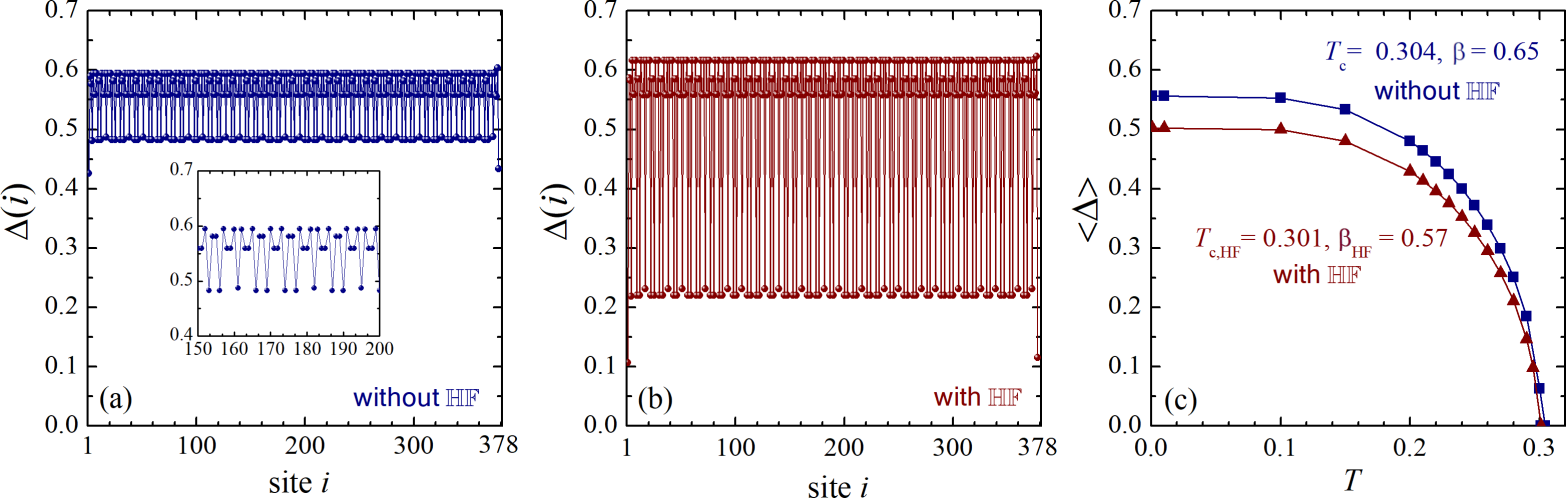
The same as in Figure 1 but for the Fibonacci chain with *n* = 13 and *t*_A_ = 0.5. Here, the critical temperatures of the systems with and without HF interaction are only slightly different: *T*_c_ = 0.304 and *T*_c,HF_ = 0.301. However, the order-parameter critical exponent for the case with the HF potential, β_HF_ = 0.57, is notably smaller than the value of β = 0.65 for the chain without the HF interaction.

For a further illustration, we consider a numerical solution of the BdG equations for a different parametric set, that is, for *n* = 13 and *t*_A_ = 0.5 (all other microscopic parameters are the same). In this case *F_n=13_* = 377 and *N* = 378. The corresponding results are shown in Figure 2. This figure shows the order-parameter spatial distribution without (Figure 2a) and with HF interaction (Figure 2b), calculated for zero temperature. Similarly to the previous case, one observes significant oscillations of the order parameter, and these oscillations are notably enhanced when including HF interaction. The maximal difference between the order-parameter minima and maxima in Figure 2a is about 20% of the spatially averaged order parameter. In Figure 2b this values becomes about 60%.

However, despite a significant enhancement of the spatial oscillations of the order parameter in the presence of the HF interaction, its spatially averaged value (in the interval from *i* = 140 to *i* = 240) does not exhibit a significant drop and is reduced by less than 10%. An even smaller difference is observed between the two critical temperatures, *T*_c_ = 0.304 and *T*_c,HF_ = 0.301. In addition, for the present case, we have ⟨Δ⟩*_T=0_*_,HF_/*T*_c,HF_ = 1.82, which is larger than the corresponding ratio without the HF field, ⟨Δ⟩*_T=0_*/*T*_c_ = 1.67. In this case, the BCS value of the ratio between the zero-temperature order parameter and the critical temperature is 1.76, that is, between the two values calculated for the Fibonacci approximant. Finally, the critical order-parameter exponents for the system with HF interaction, β_HF_ = 0.57, and without HF interaction, β = 0.65, are still larger than the corresponding BCS value of 0.5; yet, this difference is less pronounced than for the previous parametric choice.

## Conclusion

Based on a numerical solution of the BdG equations for superconducting Fibonacci chains, we demonstrate that including the HF interaction significantly enhances the spatial oscillations of the order parameter when the averaged electron density is beyond the half-filling regime. These oscillations are a direct consequence of the system’s quasiperiodicity, reflecting a general feature of superconducting quasicrystals. The enhancement of these oscillations leads to a reduction of the critical temperature, which can be pronounced depending on the model’s microscopic parameters. We also find that the critical exponent β of the order parameter differs significantly from that of a uniform BCS condensate. Moreover, the value of β changes when the HF interaction is included. Finally, the ratio between the zero-temperature order parameter and the critical temperature is also sensitive to the HF potential and deviates notably from the universal BCS value. Consequently, a rigorous analysis of the condensate distribution in quasicrystalline superconductors requires a direct comparison of results with and without HF interaction.

Finally, we remark that our results are obtained in the regime beyond the half-filling. The half-filling is the special regime with uniform density of electrons so that the HF field appears to be just a shift of the chemical potential, not altering other thermodynamic quantities, see the discussion in [22].

## Data Availability

Data generated and analyzed during this study is available from the corresponding author upon reasonable request.
